# B7-H3 as a Reliable Diagnostic Biomarker for the Differentiation of High-Grade Gliomas (HGGs) and Low-Grade Gliomas (LGGs)

**DOI:** 10.3390/brainsci15080872

**Published:** 2025-08-15

**Authors:** Fatima Juković-Bihorac, Slaviša Đuričić, Emir Begagić, Hakija Bečulić, Alma Efendić, Semir Vranić, Mirza Pojskić

**Affiliations:** 1 Department of Pathology, Cantonal Hospital Zenica, Crkvice 67, 72000 Zenica, Bosnia and Herzegovina; 2 Department of Pathology, Faculty of Medicine, University of Zenica, Travnička 1, 72000 Zenica, Bosnia and Herzegovina; slavisa.djuricic@gmail.com; 3 Department of Pathology, Faculty of Medicine, University of Banja Luka, Save Mrkalja 14, 78000 Banja Luka, Bosnia and Herzegovina; 4 Department of Neurosurgery, Cantonal Hospital Zenica, Crkvice 67, 72000 Zenica, Bosnia and Herzegovina; dr_beculichakija@hotmail.com; 5 Department of Anatomy, Faculty of Medicine, University of Zenica, Travnička 1, 72000 Zenica, Bosnia and Herzegovina; 6 Department of Radiology, Faculty of Medicine, University of Zenica, Travnička 1, 72000 Zenica, Bosnia and Herzegovina; alma_efendic@yahoo.com; 7 Department of Radiology, Medical Institute Bayer, Alekse Šantića 8, 75000 Tuzla, Bosnia and Herzegovina; 8 Department of Pathology, College of Medicine, QU Health, Qatar University, Doha P.O. Box 2713, Qatar; semir.vranic@gmail.com; 9 Department of Neurosurgery, University of Marburg, 35037 Marburg, Germany

**Keywords:** brain neoplasms, gliomas, low-grade gliomas, high-grade gliomas, B7-H3 protein, immunohistochemistry, prognosis

## Abstract

**Background/Objectives:** This study aimed to evaluate the diagnostic and prognostic utility of B7-H3 expression in differentiating low-grade gliomas (LGGs) from high-grade gliomas (HGGs) and to examine its association with clinical outcomes. **Methods:** This retrospective study included 99 patients with histopathologically confirmed gliomas (42 LGGs and 57 HGGs). B7-H3 expression was assessed using immunohistochemistry and scored by immunoreactive score (IRS). **Results:** B7-H3 expression was significantly higher in HGG compared to LGG (*p* < 0.001). The total IRS (B7-H3 A × B) demonstrated strong discriminative power (AUC = 0.816). High B7-H3 expression independently predicted disease progression (OR = 4.9, 95% CI: 2.4–10.1; *p* < 0.001) and was associated with IDH wild-type status and elevated Ki-67 index. Patients with high B7-H3 had significantly shorter overall survival (median 6 months vs. 42 months) and progression-free survival (median 3 months vs. 25 months) (both *p* < 0.001). Cox regression confirmed high B7-H3 as an independent predictor of mortality (HR = 2.9, 95% CI: 1.7–4.7; *p* < 0.001) and progression (HR = 2.6, 95% CI: 1.6–4.2; *p* < 0.001). **Conclusions:** B7-H3 expression is a reliable biomarker for distinguishing HGG from LGG and is independently associated with worse survival outcomes. Its assessment may aid in glioma classification and prognostication.

## 1. Introduction

Gliomas are tumors arising from glial cells and represent the majority of primary malignant brain tumors in adults [1]. Low-grade gliomas (LGGs) are comparatively uncommon (e.g., grade 2 gliomas account for ~6% of adult CNS tumors) and generally exhibit a more indolent course, whereas high-grade gliomas (HGGs)—particularly glioblastoma (GBM, WHO grade 4)—are aggressive [2]. For example, GBM comprises ~57% of all gliomas and nearly half of adult CNS malignancies, with a median survival of <two years. This grade distinction has crucial prognostic and therapeutic implications, yet pre-treatment differentiation of LGG versus HGG remains challenging in the clinic [2,3].

B7-H3 (CD276) is an immunoregulatory glycoprotein in the B7 family. It functions as an immune checkpoint that usually inhibits T-cell activation and proliferation, thereby facilitating tumor immune evasion [4,5]. Unlike other B7 ligands, the cognate receptor for B7-H3 is not well-defined, reflecting its complex biology [6]. Importantly, B7-H3 has limited expression in normal tissues but is overexpressed in many malignancies [7]. High tumor-associated B7-H3 levels are linked to enhanced proliferation, invasion, and worse clinical outcomes. These features have spurred interest in B7-H3 as a cancer immunotherapy target [8].

B7-H3 is frequently upregulated in gliomas [9]. Transcriptomic analyses (TCGA/CGGA) show that glioma cells express higher levels of B7-H3 mRNA than any other B7 family member. Correspondingly, ~86% of glioma specimens exhibit B7-H3 protein by immunohistochemistry, often at moderate-to-high intensity [10,11,12]. B7-H3 expression correlates well with tumor grade: it is significantly higher in GBM and IDH-wildtype gliomas, and high B7-H3 levels predict poorer survival. Mechanistic studies indicate that B7-H3 promotes glioma cell proliferation and invasion via the JAK2/STAT3/Slug pathway and by inducing epithelial–mesenchymal transition (downregulating E-cadherin and upregulating MMP-2/9) [13,14,10]. Beyond diffuse gliomas, high B7-H3 is also seen in other CNS tumors: diffuse intrinsic pontine gliomas (DIPGs), atypical teratoid/rhabdoid tumors (ATRT), neuroblastomas, medulloblastomas, ependymomas, craniopharyngiomas, and meningiomas. These cancers typically show moderate-to-strong B7-H3 expression. Notably, all ATRT cases express B7-H3 (with >90% strongly positive), and 75–100% of meningiomas are B7-H3-positive [15]. Because of its widespread overexpression, B7-H3 is being actively pursued as an immunotherapeutic target: anti–B7–H3 CAR-T-cells and antibody–drug conjugates have shown anti-tumor activity in preclinical models of GBM, DIPG, and other pediatric brain tumors [16,17,18,19,20].

Given the dismal prognosis of HGG, reliable biomarkers that distinguish HGG from LGG are urgently needed to improve diagnosis and guide treatment [21,22]. However, current diagnostic methods have limitations. For example, conventional magnetic resonance imaging (MRI) cannot reliably discriminate HGG from LGG, and biopsy sampling may miss regions of higher-grade disease. Established molecular markers (e.g., IDH mutation, 1p/19q codeletion) improve classification, but additional grade-specific markers could further refine diagnosis [23,24,25]. In this context, a robust tissue or liquid biomarker of glioma grade would complement existing tools and enable more accurate prognostication and treatment planning.

In this study, we investigate the potential utility of B7-H3 as a diagnostic biomarker to differentiate LGG from HGG. We hypothesize that, owing to its elevated expression in HGG, B7-H3 immunostaining could serve as a reliable adjunct for glioma grading. The present work aims to evaluate the utility of B7-H3 expression in distinguishing LGG versus HGG.

## 2. Materials and Methods

### 2.1. Study Design and Patients

This retrospective study included 99 consecutive patients with confirmed intracranial adult-type gliomas (LGGs and HGGs) who underwent surgical treatment at the Department of Neurosurgery, Cantonal Hospital Zenica, between 1 January 2013 and 31 December 2021. Patients with multiple primary intracranial tumors, hemorrhage, certain incidental intracranial tumors, metastatic cancers to the brain, a history of other malignancies, incomplete follow-up data, or a lack of representative tissue samples were excluded.

Patients were divided into two groups: the LGG group, including grades 1 and 2, and the HGG group, including grades 3 and 4.

### 2.2. Methods

#### 2.2.1. Histopathologic and Immunohistochemical Analysis

Following surgical excision, tumor samples were fixed in 10% neutral buffered formalin, dehydrated in ascending concentrations of ethyl alcohol (70%, 96%, 100%), and embedded in paraffin blocks. All tumor specimens were stained with hematoxylin and eosin (H&E) and graded based on histopathologic assessment at the Department of Pathology, Cantonal Hospital Zenica, or at referral centers for consultative biopsies. New serial sections with a thickness of 4 µm were prepared from paraffin blocks using a rotary microtome (microTec CUT 4055, Mainz, Germany). For repeated histopathologic and immunohistochemical analyses, sections were deparaffinized in xylene, rehydrated through descending concentrations of ethyl alcohol (100%, 96%, 70%), and rinsed in distilled water. H&E staining was performed following the standard protocol using an automated stainer (ST 4040, Leica Microsystems, Wetzlar, Germany). The prepared slides were analyzed using a light microscope (Leica DM 2500, Wetzlar, Germany).

Immunohistochemical analysis was performed on representative samples of primary tumors selected from H&E-stained slides. All sections (4 µm) were processed on an automated immunostainer (BenchMark ULTRA, Ventana Medical Systems, Inc., Tucson, AZ, USA). Membranous B7-H3 protein was detected with a validated anti-B7-H3 antibody, and expression levels were compared with recorded glioma morphology, histological type and grade, postoperative complications, and patient survival. IDH mutational status was assessed on serial sections with the mutation-specific monoclonal antibody anti-IDH1 R132H (clone H09, Dianova, Lausanne, Switzerland) on the same platform, using heat-induced epitope retrieval (Cell Conditioning 1, 32 min), primary-antibody incubation (1:50, 16 min), and OptiView DAB detection. B7-H3 staining intensity was scored with the immunoreactive score described by Meyerholz et al. [26]. The tonsils served as a positive control for B7-H3, while normal thyroid tissue was used as a negative control [27,28].

The scoring system is based on a two-category assessment. Category A refers to the evaluation of immunostaining intensity, scored as follows: 0—no staining (negative); 1—weak staining; 2—moderate staining; and 3—strong staining. Category B represents the percentage of immunoreactive cells and is scored according to the following criteria: 0—negative expression (no positive cells or only one positive cell); 1—low expression (fewer than 10% positive cells); 2—moderate expression (10–50% positive cells); and 3—high expression (more than 50% positive cells). After evaluation, the scores from Category A and Category B are multiplied to yield a final immunoreactive score (IRS) with a maximum value of 12, as described by Gansen et al. [29]. The assessment was conducted by two independent pathologists using blind samples. In cases of discrepancies, a discussion was held, and a consensus decision regarding the IRS assessment was reached jointly.

**Figure 1:**
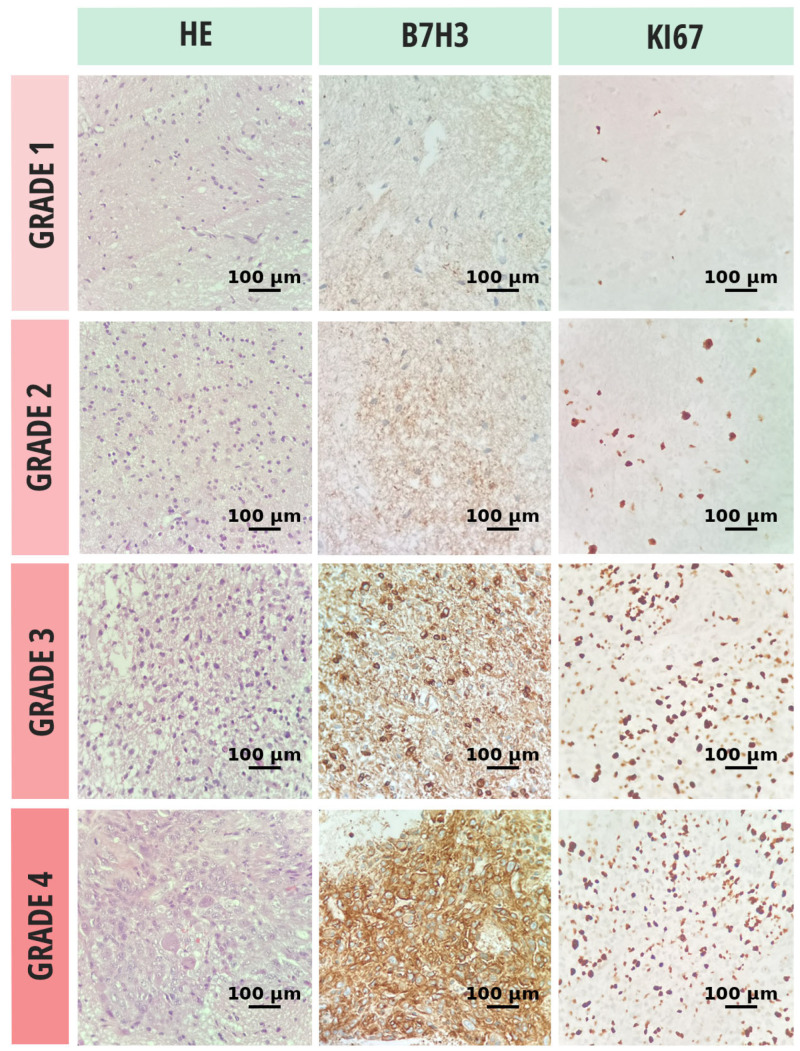
Histopathologic evaluation of glioma tissue samples, stratified by tumor grade, included hematoxylin and eosin (H&E) staining for morphological classification, immunohistochemical assessment of B7-H3 expression, and quantification of proliferative activity via the Ki-67 labeling index. All microscopic evaluations were performed at 20× objective magnification.

Cell proliferation and differentiation were assessed using the Ki-67 index. Antigen retrieval was performed by immersing the slides in a thermostatic water bath containing preheated 10 mmol/L citrate buffer (pH 6.0) for 40 min at 97 °C, followed by cooling at room temperature for 20 min. Subsequently, the slides were treated with 3.0% hydrogen peroxide (H_2_O_2_) in distilled water for 10 min to block endogenous peroxidase activity. After blocking nonspecific antigens with normal rabbit serum for 10 min, the slides were incubated at room temperature for 30 min with anti-human Ki-67 antibody (1:200; clone MIB-1, code M7240; Dako, Glostrup, Denmark). Only definitive nuclear staining was considered positive. Each case was scored by determining the percentage of stained tumor cells in multiple high-power fields with the highest staining intensity (hot spots) (Figure 1).

#### 2.2.2. Follow-Up

Disease progression was defined according to the RANO (Response Assessment in Neuro-Oncology) criteria. The RANO criteria classify treatment response into four main categories [30]. A complete response is characterized by the disappearance of all measurable lesions on contrast-enhanced MRI, the absence of new lesions, the discontinuation or minimal use of corticosteroids, and a stable or improved clinical status. A partial response is defined as a ≥50% reduction in the size of target lesions compared to baseline, without the appearance of new lesions, with stable or reduced corticosteroid use, and an unchanged or improved clinical condition. Stable disease is indicated when changes in tumor size do not meet the criteria for either a partial response or disease progression, with no new lesions and a stable clinical status. Disease progression is diagnosed when there is at least a 25% increase in tumor size compared to the smallest previously recorded measurement, the appearance of new lesions, clinical deterioration not attributable to other factors, or an increased need for corticosteroids due to symptom worsening. In cases where MRI findings were ambiguous, treatment was continued, and follow-up imaging was performed every four weeks. If progression was confirmed on follow-up imaging, the date of the initial suspicious MRI was considered the point of progression. Karnofsky performance scores (KPS) [31] were assessed before surgery and one month postoperatively to evaluate functional status.

Overall survival (OS) was defined as the time from diagnosis to death from any cause. Progression-free survival (PFS) was defined as the time from surgery to the occurrence of disease progression as per RANO criteria. Patients were followed for a minimum of three years from the time of brain tumor diagnosis, taking into account the natural course of the disease.

### 2.3. Statistical Analysis

Statistical analysis was conducted using SPSS (Statistical Package for the Social Sciences), version 26.0. Categorical variables were presented as frequencies and percentages. Continuous variables were reported according to the distribution of data. The normality of distribution was assessed using the Shapiro–Wilk test and Q-Q plots. Due to deviations from normal distribution, data were expressed as median and interquartile range (IQR). Statistically significant differences between groups were determined using the Mann-Whitney U test. The discriminative ability of B7-H3 expression to distinguish between LGG and HGG was evaluated using the Receiver Operating Characteristic (ROC) curve analysis. The results were presented as the area under the curve (AUC) with the corresponding 95% Confidence Interval (CI). The optimal cut-off value was determined using the Youden index. To assess the predictive role of the investigated marker, univariate regression analysis was performed. Variables that demonstrated statistical significance in univariate analysis were further analyzed using multivariate regression. The results of the regression analyses were presented as odds ratios (OR). The predictive value of B7-H3 expression for overall survival (OS) and progression-free survival (PFS) was assessed using Cox proportional hazards regression, with results presented as hazard ratios (HR). The level of statistical significance was set at *p* ≤ 0.05.

## 3. Results

### 3.1. Sociodemographic and Baseline Characteristics

Table 1 presents the demographic characteristics of the entire study (N = 99) and by groups: LGG (N = 42) and HGG (N = 57). Among the patients, 57 (57.6%) were male, with a higher proportion in the HGG group (63.2%) compared to the LGG group (50.0%), although this difference was not statistically significant (*p* = 0.190). Similarly, 42 (42.4%) participants were female, equally represented in the LGG group (50.0%) but less so in the HGG group (36.8%). The median age in the entire cohort was 56 years (IQR 46–63), with medians of 55 years (IQR 44–65) in the LGG group and 56 years (IQR 47–63) in the HGG group, respectively. Age differences between the groups were not statistically significant (*p* = 0.899).

**Figure 2:**
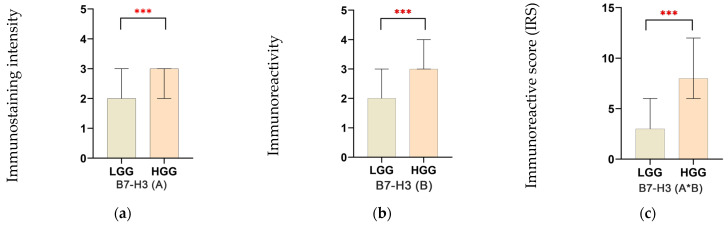
Assessment of immunostaining intensity (**a**), immunoreactivity of neoplastic glial cells (**b**), and composite immunoreactive score for B7-H3 expression (**c**). Data are shown as median with interquartile range (IQR). *** *p* < 0.001.

The intensity of B7-H3 immunostaining (B7-H3(A)) had a median value of 2.0 (IQR 1.0–3.0) in the LGG group, compared to a higher median of 3.0 (IQR 2.0–6.0) in the HGG group (*p* < 0.001) (Figure 2a). When assessing the percentage of immunoreactive glial neoplastic cells for B7-H3 (B7-H3(B)), the median value for LGG was 2.0 (IQR 2.0–3.0) (Figure 2b), whereas the HGG group showed significantly higher median values of 3.0 (IQR 3.0–4.0) (Figure 2c), with a statistically significant difference (*p* < 0.001).

Additionally, the Ki-67 proliferation index was significantly higher in the HGG group (median 30%, 95% CI: 25.0–60.0) compared to the LGG group (median 5%, 95% CI: 4.0–20.0) (*p* < 0.001), indicating increased tumor cell proliferation in HGG.

### 3.2. Discriminative Role of B7-H3 in Glial Neoplasms

**Figure 3:**
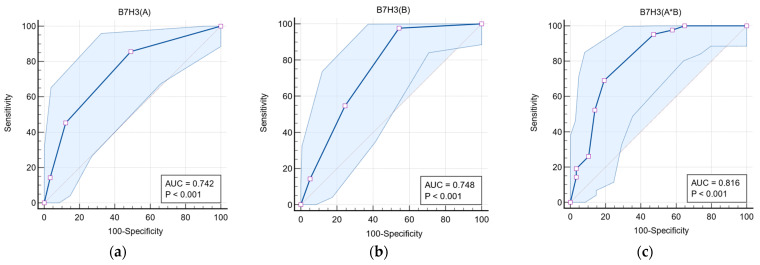
Receiver Operating Characteristic (ROC) analysis of the discriminative value of immunostaining intensity (**a**), immunoreactivity (**b**), and the total immunoreactive expression score (**c**) of B7-H3 in differentiating high-grade from low-grade glial tumors.

Table 2 presents the discriminative role of B7-H3 expression in differentiating LGG from HGG tumors. For the variable B7-H3 (A), at a cut-off value of ≥2, the area under the curve (AUC) was 0.742 (95% CI: 0.645–0.825) (Figure 3a), with a sensitivity of 85.71% and specificity of 50.88%. For B7-H3 (B), at a cut-off value of ≥3, the AUC was 0.748 (95% CI: 0.651–0.830) (Figure 3b), with a sensitivity of 97.62% and specificity of 45.61%. For the total immunoexpression score, B7-H3 (A*B), at a cut-off value of ≥5, the AUC was 0.816 (95% CI: 0.726–0.886), as shown in Figure 3c, with a sensitivity of 73.14% and specificity of 80.70%.

**Figure 4:**
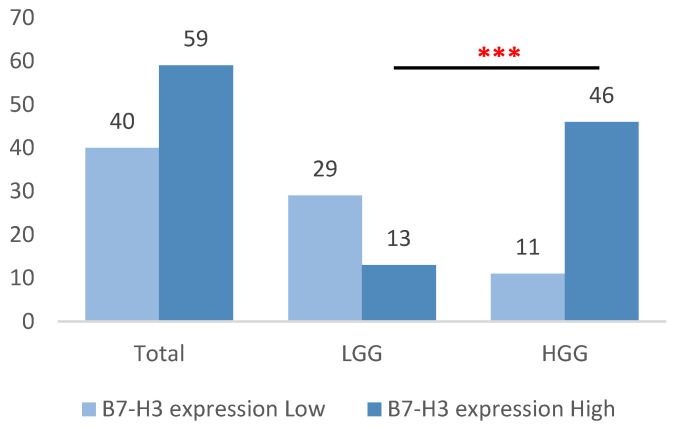
Distribution of high and low B7-H3 expression among HGG and LGG tumors, classified according to cut-off values established by ROC analysis. ***, statistically significant difference (*p* < 0.001).

Based on previously defined cut-off values, low B7-H3 expression was observed in 40 samples (40.4%) overall, predominantly in the LGG group (29 samples, 69.0%), in contrast to only 11 samples in the HGG group (19.3%). Conversely, high B7-H3 expression was present in 59 samples (59.6%), with a markedly higher frequency in the HGG group (46 samples, 80.7%) compared to the LGG group (13 samples, 31.0%) (*p* < 0.001) (Figure 4).

### 3.3. Follow-Up Data

The preoperative KPS differs significantly between groups (Table 3). In the total sample, the median score was 40.0 (IQR 20.0–60.0). The LGG group had a higher median preoperative KPS of 60.0 (IQR 40.0–80.0), whereas the HGG group showed a significantly lower median of 40.0 (IQR 20.0–40.0) (*p* < 0.001). Postoperatively, the median KPS remained higher in the LGG group at 60.0 (IQR 40.0–100.0), compared to 20.0 (IQR 20.0–60.0) in the HGG group (*p* < 0.001). Disease assessment according to RANO criteria revealed significant differences in therapy response. A complete response was observed in four patients (4.0%), all from the LGG group. A partial response was recorded in 17 patients (17.2%), also exclusively in the LGG group. Stable disease was noted in twenty patients (20.2%), more frequently in the LGG group (fifteen patients, 35.7%) compared to the HGG group (five patients, 8.8%). Disease progression predominated in the HGG group, occurring in fifty-two patients (91.2%), whereas only six patients (14.3%) in the LGG group showed progression (*p* < 0.001).

**Figure 5:**
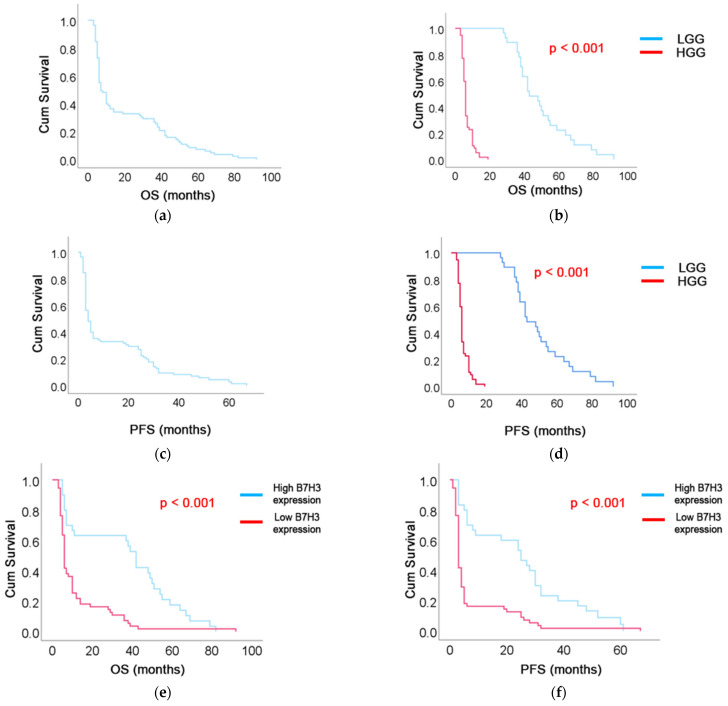
Kaplan–Meier survival curves showing (**a**) overall survival (OS) in the entire cohort, (**b**) OS stratified by tumor grade (LGG vs. HGG), (**c**) progression-free survival (PFS) in the entire cohort, (**d**) PFS by tumor grade, (**e**) OS according to B7-H3 expression level (high vs. low), and (**f**) PFS according to B7-H3 expression level.

The median OS in the entire cohort was 10 months (95% CI: 5–37) (Figure 5a). OS was significantly extended in patients with LGG, with a median of 43 months (IQR: 38–57), compared to six months (IQR: 4–10) in the HGG group (*p* < 0.001) (Figure 5b). PFS in the entire cohort was seven months (5–28) (Figure 5c). PFS was notably prolonged in the LGG group, with a median of 30 months (IQR: 25–42), compared to three months (IQR: 3–4) in the HGG group (*p* < 0.001) (Figure 5d).

The median OS in the high B7-H3 expression group was six months (95% CI: 5.4–6.6), whereas in the low expression group it was significantly longer at 42 months (95% CI: 36.9–47.1) (*p* < 0.001), as shown in Figure 5e. The median PFS in the high B7-H3 expression group was three months (95% CI: 2.6–3.4), compared to 25 months (95% CI: 15.4–34.6) in the low expression group. This difference was statistically significant (*p* < 0.001) (Figure 5f).

### 3.4. Predictive Value of B7-H3 Expression in Glial Neoplasms

Univariate regression analysis demonstrated that high B7-H3 expression was associated with a greater likelihood of poor clinical status both preoperatively and one month postoperatively, as well as with disease progression. Preoperatively, patients with high B7-H3 expression were 4.3 times more likely to have a KPS below 60 (95% CI: 1.8–10.4; *p* < 0.001), and this likelihood slightly increased to 4.5 times one month after surgery (95% CI: 1.9–10.6; *p* < 0.001). Multivariate regression analysis confirmed that patients with high B7-H3 expression had a 4.9-fold increased likelihood of disease progression according to the RANO criteria (95% CI: 2.4–10.1; *p* < 0.001), even after adjusting for other clinical and molecular factors. Additionally, high B7-H3 expression was significantly associated with IDH wild-type status in the univariate analysis (OR = 3.8; 95% CI: 1.6–8.9; *p* = 0.002), and this association remained significant in the multivariate model (OR = 3.4; 95% CI: 1.3–8.5; *p* = 0.011). Similarly, a Ki-67 index > 25% was strongly associated with high B7-H3 expression (univariate OR = 4.6; 95% CI: 2.1–10.2; *p* < 0.001; multivariate OR = 3.7; 95% CI: 1.5–9.0; *p* = 0.005), suggesting a link between B7-H3 expression and biologically aggressive tumor behavior. In contrast, neither age > 60 years (OR = 2.1; 95% CI 0.9–4.9; *p* = 0.083) nor male sex (OR = 1.2; 95% CI 0.7–2.2; *p* = 0.521) was significantly associated with high B7-H3 expression, indicating that these basic demographic factors were not major determinants of B7-H3 expression (Table 4).

Cox regression analysis revealed that high B7-H3 expression was significantly associated with shorter OS and PFS in patients with glial neoplasms. Specifically, patients with high B7-H3 expression had a 2.9-fold increased risk of mortality (95% CI: 1.7–4.7; *p* < 0.001) compared to those with low expression. The risk of disease progression was also 2.6 times higher (95% CI: 1.6–4.2; *p* < 0.001) in patients with high B7-H3 expression, suggesting that it may serve as a potential adverse prognostic marker in glial neoplasms.

## 4. Discussion

Our study is, to our knowledge, the first to assess the diagnostic accuracy (sensitivity and specificity) of B7-H3 immunoreactivity for differentiating high- from low-grade glioma, and it further demonstrates that elevated B7-H3 expression correlates with higher tumor grade and poorer clinical outcomes (reduced OS and PFS).

The differential expression of B7-H3 protein across glioma grades underscores its potential as a diagnostic marker. Our findings are consistent with previous studies that have reported elevated B7-H3 expression in higher-grade gliomas. For instance, a study by Zhong et al. [32] demonstrated that B7-H3 expression was significantly higher in HGG tissues compared to LGG counterparts, suggesting its involvement in tumor progression. Similarly, Digregorio et al. [33] reported that B7-H3 expression was associated with more aggressive glial neoplasms [33]. Nehama et al. [34] evaluated B7-H3 expression in GBM specimens and found that 76% displayed strong immunoreactivity, 22% (10/46) showed low expression, and only one specimen lacked B7-H3 expression entirely. These findings underscore the association between elevated B7-H3 expression and higher glioma grades.

The prognostic implications of B7-H3 expression in gliomas have been a subject of considerable interest [35]. Our study’s observation that elevated B7-H3 expression correlates with poorer OS and PFS is corroborated by multiple investigations. Elevated B7-H3 expression has been linked to poorer survival outcomes in glioma patients. In a database from Huashan Hospital of Fudan University, GBM patients with high B7-H3 expression (IRS > 6) had a median OS of 12 months, compared to 16 months in the low-expression group (IRS ≤ 6), with the difference being statistically significant (*p* = 0.042) [35]. Analysis of the Rembrandt dataset corroborated these findings, showing significantly lower OS in the high B7-H3 expression group (N = 88) compared to the low-expression group (N = 89), with a *p*-value of 0.0055 [35]. In pediatric gliomas, higher B7-H3 mRNA expression was associated with shorter median survival (13.2 months) compared to lower expression (19.2 months), with a *p*-value of 0.049, as reported by Maachani et al. [10].

Beyond its diagnostic and prognostic roles, B7-H3 appears to actively contribute to glioma pathogenesis. Mechanistically, B7-H3 has been implicated in promoting tumor cell proliferation, invasion, and immune evasion. Zhong et al. [32] demonstrated that B7-H3 overexpression in glioma cells enhanced proliferation and invasion through activation of the JAK2/STAT3/Slug signaling pathway, leading to epithelial–mesenchymal transition (EMT) and increased expression of matrix metalloproteinases. Additionally, B7-H3 has been shown to suppress natural killer (NK) cell-mediated cytotoxicity, facilitating immune escape [32]. Mechanistically, B7-H3 appears to activate key signaling pathways, including PI3K/AKT and JAK2/STAT3, which are known to support CSC maintenance and proliferation [9]. These findings suggest that B7-H3 contributes to the resilience and persistence of CSCs in gliomas [32], as well as the regulation of the growth of gliomas [32].

Given its tumor-specific expression and role in promoting malignancy, B7-H3 presents an attractive target for therapeutic intervention. Chimeric antigen receptor (CAR) T-cell therapies targeting B7-H3 have shown promise in preclinical models of glioblastoma, demonstrating potent antitumor activity and prolonged survival [36]. Moreover, antibody–drug conjugates and monoclonal antibodies directed against B7-H3 are under investigation, with early-phase clinical trials indicating favorable safety profiles and preliminary efficacy [37,11].

While our study provides compelling evidence for the clinical relevance of B7-H3 in gliomas, certain limitations warrant consideration. The retrospective nature of the study and the relatively small sample size may limit the generalizability of the findings. Future studies should aim to validate these findings in larger, prospective cohorts and explore the therapeutic efficacy of B7-H3-targeted interventions in clinical settings.

## 5. Conclusions

This study demonstrates that B7-H3 expression is significantly elevated in HGG compared to LGG and correlates with adverse pathological and clinical features, including higher proliferative activity, IDH-wildtype status, and increased risk of disease progression. Multivariate regression analysis confirmed that high B7-H3 expression is an independent predictor of glioma grade and progression, while Cox regression analysis identified it as a significant prognostic factor for both reduced overall survival and progression-free survival. These findings suggest that B7-H3 may serve as a reliable diagnostic and prognostic biomarker in glioma patients and could inform future strategies for molecular classification and targeted therapeutic approaches.

## Figures and Tables

**Table 1:** Sociodemographic and baseline characteristics of patients.

Variable		Total (N = 99)	Groups		
			LGG (N = 42)	HGG (N = 57)	*p*-Value
Demographic data					
Gender N (%)	Male	57 (57.6)	21 (50.0)	36 (63.2)	0.190
	Female	42 (42.4)	21 (50.0)	21 (36.8)	
Age (years) Median (IQR)		56 (46–63)	55 (44–65)	56 (47–63)	0.899
Histopathologic data					
Grade N (%)	G1	12 (12.1)	12 (28.6)	-	<0.001
	G2	30 (30.3)	30 (71.4)	-	
	G3	21 (21.2)	-	21 (36.8)	
	G4	36 (36.4)	-	36 (63.2)	
Ki-67 (%) Median (IQR)		20.0 (8.0–60.0)	5.0 (4.0–20.0)	30.0 (25.0–60.0)	<0.001
IDH status	IDH-mutant	55 (55.6%)	31 (73.8%)	9 (15.8%)	<0.001
	IDH-wild type	44 (44.4%)	11 (26.2%)	48 (84.2%)	

LGG—low-grade glioma; HGG—high-grade glioma; N—frequency; IQR—interquartile range.

**Table 2:** Receiver Operating Characteristic (ROC) analysis evaluating the discriminative role of B7-H3 in distinguishing between LGG and HGG neoplasms.

Variable	AUC	95% CI	Cut-Off	Sensitivity	Specificity	*p*-Value
B7-H3 (A)	0.742	0.645–0.825	≥2	85.71	50.88	<0.001
B7-H3 (B)	0.748	0.651–0.830	≥3	97.62	45.61	<0.001
B7-H3 (A*B)	0.816	0.726–0.886	≥5	73.14	80.70	<0.001

**Table 3:** Follow-up data for the glioma cohort.

Variable		Total (N = 99)	Groups		
			LGG (N = 42)	HGG (N = 57)	*p*-Value
KPS preoperatively Median (IQR)		40.0 (20.0–60.0)	60.0 (40.0–80.0)	40.0 (20.0–40.0)	<0.001
KPS first follow-up (1 month) Median (IQR)		40.0 (20.0–100.0)	60.0 (40.0–100.0)	20.0 (20.0–60.0)	<0.001
Overall survival (months) Median (IQR)		10 (5–37)	43 (38–57)	6 (4–10)	<0.001
RANO criteria of disease progression	Complete response	4 (4.0)	4 (9.5)	0 (0)	<0.001
	Partial response	17 (17.2)	17 (40.5)	0 (0)	
	Stable disease	20 (20.2)	15 (35.7)	5 (8.8)	
	Progression	58 (58.6)	6 (14.3)	52 (91.2)	
PFS (months) Median (IQR)		7 (5–28)	30 (25–42)	3 (3–4)	<0.001

**Table 4:** Univariate and multivariate logistic regression analysis of factors associated with high B7-H3 expression in glioma patients.

Variable	Univariate Regression Analysis		Multivariate Regression Analysis	
	OR (95% CI)	*p*	OR (95% CI)	*p*-Value
Age > 60 years	2.1 (0.9–4.9)	0.083	-	-
Gender (male)	1.2 (0.7–2.2)	0.521	-	-
KPS preoperatively < 60	4.3 (1.8–10.4)	<0.001	1.7 (0.1–29.4)	0.718
KPS postoperatively < 60	4.5 (1.9–10.6)	<0.001	2.6 (0.2–44.7)	0.500
RANO criteria = progression	5.4 (2.7–10.6)	<0.001	4.9 (2.4–10.1)	<0.001
IDH wild type	3.8 (1.6–8.9)	0.002	3.4 (1.3–8.5)	0.011
Ki-67 > 25%	4.6 (2.1–10.2)	<0.001	3.7 (1.5–9.0)	0.005

KPS—Karnofsky performance status; OR—odds ratio; CI—confidence interval.

## Data Availability

The original contributions presented in this study are included in the article. Further inquiries can be directed at the corresponding authors.
